# Supplementary material for: On the relationship between emotions and cognitive control: Evidence from an observational study on emotional priming Stroop task
Source: PLoS One. 2023 Nov 27;18(11):e0294957. doi: 10.1371/journal.pone.0294957 (PMC10681184; doi:10.1371/journal.pone.0294957)
Supplement: S1 Checklist — (DOCX) [file pone.0294957.s001.docx]

STROBE Statement—checklist of items that should be included in reports of observational studies

|  | Item No. | Recommendation | Page  No. | Relevant text from manuscript |
| --- | --- | --- | --- | --- |
| **Title and abstract** | 1 | (*a*) Indicate the study’s design with a commonly used term in the title or the abstract | 1 | “Evidence from an observational study on emotional priming Stroop task” |
|  |  | (*b*) Provide in the abstract an informative and balanced summary of what was done and what was found | 1 | “This observational study sought to examine this interaction by looking at the distinction between proactive and reactive modes of control and how they relate with emotional processing. Seventy-four healthy participants performed an emotional priming Stroop task. On each trial, target stimuli of a spatial Stroop task were preceded by sad or neutral facial expressions, providing two emotional conditions. To manipulate the requirement of both proactive and reactive control, the proportion of congruent trials (PC) was varied at the list-wide (LWPC) and item-specific (ISPC) levels, respectively. We found that sad priming led to behavioral costs only in trials with low proactive and reactive cognitive control exertion.” |
| Introduction | | | |  |
| Background/rationale | 2 | Explain the scientific background and rationale for the investigation being reported | 2-5 | Pages 2-5 |
| Objectives | 3 | State specific objectives, including any prespecified hypotheses | 6 | See page 6 |
| Methods | | | |  |
| Study design | 4 | Present key elements of study design early in the paper | 6-11 | Pages 6-11 |
| Setting | 5 | Describe the setting, locations, and relevant dates, including periods of recruitment, exposure, follow-up, and data collection | 6-7 | Pages 6-7 |
| Participants | 6 | (*a*) *Cohort study*—Give the eligibility criteria, and the sources and methods of selection of participants. Describe methods of follow-up  *Case-control study*—Give the eligibility criteria, and the sources and methods of case ascertainment and control selection. Give the rationale for the choice of cases and controls  *Cross-sectional study*—Give the eligibility criteria, and the sources and methods of selection of participants | NA, our study was an observational study on healthy participants (not a clinical study) |  |
|  |  | (*b*) *Cohort study*—For matched studies, give matching criteria and number of exposed and unexposed  *Case-control study*—For matched studies, give matching criteria and the number of controls per case | NA | NA |
| Variables | 7 | Clearly define all outcomes, exposures, predictors, potential confounders, and effect modifiers. Give diagnostic criteria, if applicable | NA | NA |
| Data sources/ measurement | 8* | For each variable of interest, give sources of data and details of methods of assessment (measurement).  Describe comparability of assessment methods if there is more than one group | See methods section  NA |  |
| Bias | 9 | Describe any efforts to address potential sources of bias | NA |  |
| Study size | 10 | Explain how the study size was arrived at | Page 7 | Page 7, paragraph starting with: “The method introduced by Westfall and colleagues (36) was used to perform a power analysis for a fully-crossed linear mixed-effects model, assuming participant and stimulus intercepts, participant slope, and residual variance partitioning coefficients of .1, .1, .2, and .6, respectively, as estimated conservatively from some recent unpublished studies with a similar design from our research group…etc.” |

Continued on next page

| Quantitative variables | 11 | Explain how quantitative variables were handled in the analyses. If applicable, describe which groupings were chosen and why | Pages 11-12 | See Paragraph on Statistical analyses starting with “Analyses of RTs were performed by means of linear mixed-effects models (LMM)…” |
| --- | --- | --- | --- | --- |
| Statistical methods | 12 | (*a*) Describe all statistical methods, including those used to control for confounding | Pages 11-12 | See Paragraph on Statistical analyses starting with “Analyses of RTs were performed by means of linear mixed-effects models (LMM)…” |
|  |  | (*b*) Describe any methods used to examine subgroups and interactions | NA | NA |
|  |  | (*c*) Explain how missing data were addressed | NA | NA |
|  |  | (*d*) *Cohort study*—If applicable, explain how loss to follow-up was addressed  *Case-control study*—If applicable, explain how matching of cases and controls was addressed  *Cross-sectional study*—If applicable, describe analytical methods taking account of sampling strategy | NA | NA |
|  |  | (*e*) Describe any sensitivity analyses | NA | NA |
| Results | | | | |
| Participants | 13* | (a) Report numbers of individuals at each stage of study—eg numbers potentially eligible, examined for eligibility, confirmed eligible, included in the study, completing follow-up, and analysed | NA. Not possible to know (all healthy young people using internet were potentially eligible) | NA |
|  |  | (b) Give reasons for non-participation at each stage | NA | NA |
|  |  | (c) Consider use of a flow diagram | NA | NA |
| Descriptive data | 14* | (a) Give characteristics of study participants (eg demographic, clinical, social) and information on exposures and potential confounders | Page 6 | Page 6: “Seventy-four participants completed the task online between September 2021 and January 2022 (44 females, 28 males; mean age = 24.3 years, SD = 4.8; two participants did not provide age and sex information; 64 right-handed).” |
|  |  | (b) Indicate number of participants with missing data for each variable of interest | None | None |
|  |  | (c) *Cohort study*—Summarise follow-up time (eg, average and total amount) | NA | NA |
| Outcome data | 15* | *Cohort study*—Report numbers of outcome events or summary measures over time | NA | NA |
|  |  | *Case-control study—*Report numbers in each exposure category, or summary measures of exposure | NA | NA |
|  |  | *Cross-sectional study—*Report numbers of outcome events or summary measures | NA | NA |
| Main results | 16 | (*a*) Give unadjusted estimates and, if applicable, confounder-adjusted estimates and their precision (eg, 95% confidence interval). Make clear which confounders were adjusted for and why they were included | Pages 13-14 | Table 1 |
|  |  | (*b*) Report category boundaries when continuous variables were categorized | NA | NA |
|  |  | (*c*) If relevant, consider translating estimates of relative risk into absolute risk for a meaningful time period | NA | NA |

Continued on next page

| Other analyses | 17 | Report other analyses done—eg analyses of subgroups and interactions, and sensitivity analyses | NA | NA |
| --- | --- | --- | --- | --- |
| Discussion | | | | |
| Key results | 18 | Summarise key results with reference to study objectives | See page 15 | “Our results showed that sad emotional priming was associated with behavioral costs only in trials with low cognitive control exertion, that is, in congruent trials (where there is no need of reactive control) with lower proactive control.” |
| Limitations | 19 | Discuss limitations of the study, taking into account sources of potential bias or imprecision. Discuss both direction and magnitude of any potential bias | Page 17 | Paragraph starting with “Some caveats to our results along with some study limitations need to be acknowledged. First, our conclusions are limited to sadness…” |
| Interpretation | 20 | Give a cautious overall interpretation of results considering objectives, limitations, multiplicity of analyses, results from similar studies, and other relevant evidence | Page 17 | Paragraph starting with “Some caveats to our results along with some study limitations need to be acknowledged. First, our conclusions are limited to sadness…” |
| Generalisability | 21 | Discuss the generalisability (external validity) of the study results | Page 17 | Parts of paragraph starting with “First, our conclusions are limited to sadness, thus, further studies are needed to generalize them to emotions…” |
| Other information | |  | | |
| Funding | 22 | Give the source of funding and the role of the funders for the present study and, if applicable, for the original study on which the present article is based | Pages 17-18 | Paragraph starting with “This study was in part supported by the…” |

*Give information separately for cases and controls in case-control studies and, if applicable, for exposed and unexposed groups in cohort and cross-sectional studies.

**Note:** An Explanation and Elaboration article discusses each checklist item and gives methodological background and published examples of transparent reporting. The STROBE checklist is best used in conjunction with this article (freely available on the Web sites of PLoS Medicine at http://www.plosmedicine.org/, Annals of Internal Medicine at http://www.annals.org/, and Epidemiology at http://www.epidem.com/). Information on the STROBE Initiative is available at www.strobe-statement.org.
