# Supplementary material for: On the relationship between emotions and cognitive control: Evidence from an observational study on emotional priming Stroop task
Source: PLoS One. 2023 Nov 27;18(11):e0294957. doi: 10.1371/journal.pone.0294957 (PMC10681184; doi:10.1371/journal.pone.0294957)
Supplement: S1 Appendix — (PDF) [file pone.0294957.s002.pdf]

S1 APPENDIX. List of images used from the NimStim set of facial expressions.

01F_NE_C, 01F_SA_C, 03F_NE_O, 03F_SA_O, 07F_NE_C, 07F_SA_C, 09F_NE_C, 09F_SA_C, 10F_NE_O, 10F_SA_O, 13F_NE_O, 13F_SA_O, 14F_NE_O, 14F_SA_O, 17F_NE_C, 17F_SA_C, 18F_NE_C, 18F_SA_C, 19F_NE_C, 19F_SA_C, 20M_NE_C, 20M_SA_C, 26M_NE_C, 26M_SA_C, 27M_NE_C, 27M_SA_C, 29M_NE_C, 29M_SA_C, 30M_NE_C, 30M_SA_C, 34M_NE_C, 34M_SA_C, 39M_NE_C, 39M_SA_C, 40M_NE_C, 40M_SA_C, 43M_NE_C, 43M_SA_C, 45M_NE_O, and 45M_SA_O).
