# Supplementary material for: On the relationship between emotions and cognitive control: Evidence from an observational study on emotional priming Stroop task
Source: PLoS One. 2023 Nov 27;18(11):e0294957. doi: 10.1371/journal.pone.0294957 (PMC10681184; doi:10.1371/journal.pone.0294957)
Supplement: S1 File — (PDF) [file pone.0294957.s003.pdf]

COMITATO ETICO DELLA RICERCA PSICOLOGICA (AREA 17)
Dipartimenti/Sezione di Psicologia - Università di Padova
Via Venezia 8, 35131, Padova
FAX. +39-0498276600, Email: comitato.etico.area17@unipd.it; Sito WEB: http://ethos.psy.unipd.it/

Protocollo: 4187

Data: 10/05/2021

Numero Univoco: 71AA5B3AAE967C5273D2933B23889286

Scopo: Richiesta di parere

Titolo: Controllo cognitivo e emozione in un compito di Stroop

Proponente
Cognome e nome: Ambrosini Ettore
Ruolo: Associato
e-mail: ettore.ambrosini@unipd.it

Area: Psicologia generale
(se altro):

Ricercatori partecipanti: 5
Ambrosini Ettore - Associato - DPG e DNS Vallesi Antonino - Associato - DNS Visalli Antonino - Assegnista - DNS Sambataro Fabio - Associato - DNS Tenconi Elena - Associato - DNS

Il Comitato Etico, dopo attento esame delle informazioni fornite dal proponente, esprime parere positivo riguardante gli aspetti etici del progetto.

The project has been approved by the Ethical Committee for the Psychological Research of the University of Padova.

Si fa presente che tale approvazione è vincolata al rispetto della normativa vigente in materia di privacy. Le vigenti leggi sulla protezione dei dati (D. Lgs 196/2003 e UE GDPR 679/2016) sono valide solo per partecipanti UE

Please note that this approval is subject to compliance with current privacy legislation. The current data protection laws (D. Lgs 196/2003 and EU GDPR 679/2016) are valid only for EU participants

Il Comitato Etico si riserva di revocare l'approvazione qualora il progetto non venisse condotto secondo quanto dichiarato nel protocollo di ricerca approvato dal Comitato.
Pertanto, modifiche in qualsiasi punto della procedura o del consenso informato dovranno essere sottoposte nuovamente all'attenzione del Comitato.

Padova, 22/05/2021

Per il Comitato Etico dell'area 17

La Presidente	Il Vicepresidente	Il Segretario

I membri
