# Supplementary material for: On the relationship between emotions and cognitive control: Evidence from an observational study on emotional priming Stroop task
Source: PLoS One. 2023 Nov 27;18(11):e0294957. doi: 10.1371/journal.pone.0294957 (PMC10681184; doi:10.1371/journal.pone.0294957)
Supplement: S2 File — (PDF) [file pone.0294957.s004.pdf]

Ethics committee of the psychological research (Area 17) Psychology Departments/Section – Università di Padova Via Venezia 8, 35131, Padova FAX. +39-0498276600, Email: comitato.etico.area17@unipd.it; Sito WEB: http://ethos.psy.unipd.it/

English translation of the Ethics Committee Approval for the project entitled: **Controllo cognitivo e emozione in un compito di Stroop** (in English: Cognitive Control and emotion in a Stroop task)

Protocol: 4187

Date: 10/05/2021

Univocal Number: 71AA5B3AAE967C5273D2933B23889286

Goal: Request of evaluation

Title: Cognitive Control and emotion in a Stroop task

Proponent
Surname & Name: Ambrosini Ettore
Role: Associate Professor
e-mail: ettore.ambrosini@unipd.it

Area: General Psychology

Participant researchers: 5
Ambrosini Ettore – Associate prof. - DPG & DNS Vallesi Antonino – Associate prof. - DNS Visalli Antonino – post-doc - DNS Sambataro Fabio – Associate prof. - DNS Tenconi Elena – Associate prof. – DNS

The Ethics committee, after careful evaluation of the information provided by the proponent, expresses a positive opinion regarding the ethics aspects of the project.

The project has been approved by the Ethical Committee for the Psychological Research of the University of Padova.

Please note that this approval is subject to compliance with current privacy legislation. The current data protection laws (D. Lgs 196/2003 and EU GDPR 679/2016) are valid only for EU participants.

The Ethics committee could revoke the approval if the project would not be carried out according to what has been declared in the research protocol approved by the committee.
For this, modifications of any point of the procedure or of the informed consent will have to be submitted again to the attention of the Committee.
